# Supplementary material for: Proliferation-Based WHO Grading and Heterogeneous Gastrin Expression in Canine Gallbladder Neuroendocrine Tumors
Source: Vet Sci. 2025 Oct 14;12(10):989. doi: 10.3390/vetsci12100989 (PMC12568047; doi:10.3390/vetsci12100989)

Supplementary Table 1.

Additional Histologic Features of GB NEN (0=no, 1=yes)						
Gastrin IHC	Synaptophysin IHC	Coagulation Necrosis	Hemorrhage, Blood filled spaces	Hemosiderin	Peripheral Invasion	Large or irregular Nuclei
0	1	1	1	0	1	0
0	1	1	1	1	1	1
0	1	0	0	0	1	1
0	1	1	1	0	1	1
0	1	0	1	1	0	0
0	1	1	0	1	1	0
1	1	1	0	0	1	1
0	1	0	1	1	1	0
0	1	1	0	0	1	1
0	1	1	0	0	1	1
0	1	1	0	0	1	0
0	1	1	1	0	1	0
0	1	0	0	0	1	0
0	1	0	1	0	1	0
0	1	1	1	0	1	0
0	1	1	1	1	0	1
0	1	0	1	1	1	1
0	1	0	0	0	0	1
0	1	1	0	0	1	1
0	1	1	1	0	1	0

Supplementary Figure 1. Gastrin IHC positive control. Gastrin immunohistochemistry of formalin fixed paraffin embedded canine stomach (DAB with hematoxylin counterstain). Courtesy of the Animal Health Diagnostic Center at Cornell University College of Veterinary Medicine.

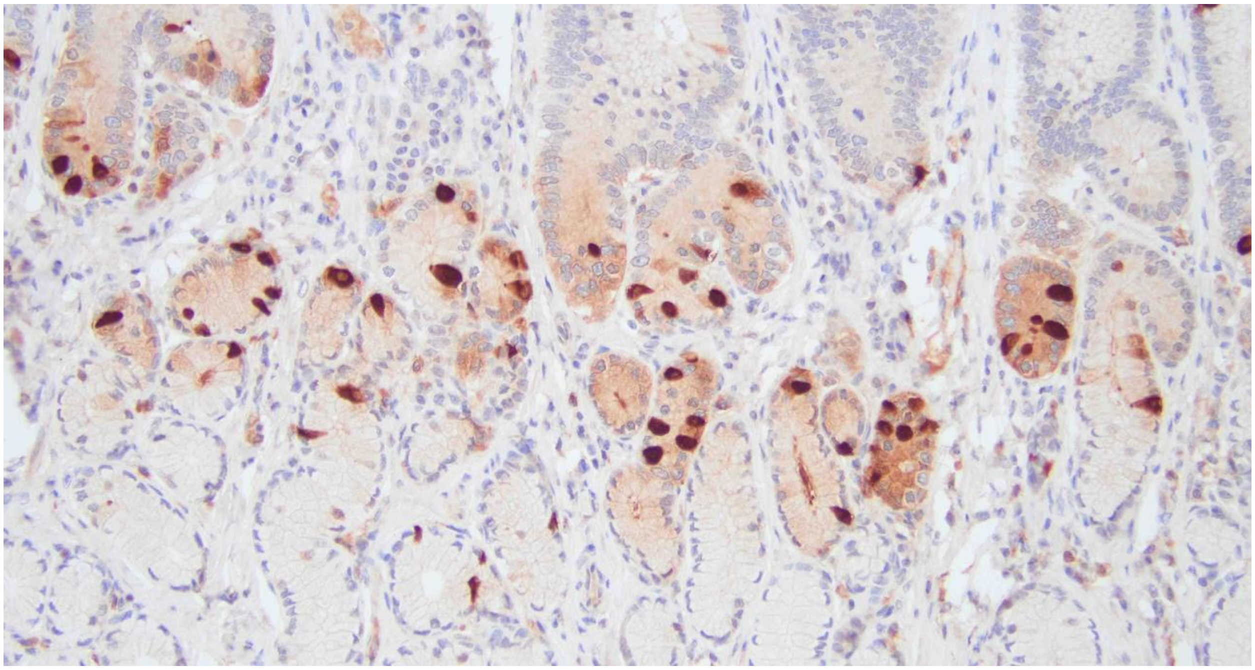

Supplement: Supplementary file 1 [file vetsci-12-00989-s001.zip › vetsci-3767747-supplementary.pdf]
